# A decentralized framework for cultivating research lifecycle transparency

**DOI:** 10.1371/journal.pone.0241496

**Published:** 2020-11-18

**Authors:** Wei Jeng, Shih-Hung Wang, Hung-Wei Chen, Po-Wei Huang, Yu-Jen Chen, Hsu-Chun Hsiao

**Affiliations:** 1 Department of Library and Information Science, National Taiwan University, Taipei, Taiwan; 2 Department of Computer Science and Information Engineering, National Taiwan University, Taipei, Taiwan; 3 Department of Computer Science, University of California, Irvine, California, United States of America; 4 Research Center for Information Technology Innovation, Academia Sinica, Taipei, Taiwan; 5 School of Public Health, Boston University, Boston, Massachusetts, United States of America; Wuhan University, CHINA

## Abstract

Research transparency has been advocated as a key means of addressing the current crisis of reproducibility. This article proposes an enhanced form of research transparency, termed lifecycle transparency. Over the entire lifecycle of a research effort, this approach captures the syntactical contexts of artifacts and stakeholders, such as timestamps, agreements, and/or dependency requirements for completing each research phase. For example, such contexts might include when, where, and from whom patients’ consent and institutional review board approvals were received before a clinical trial was carried out. However, as existing open-science tools are often dedicated to certain research phases or disciplines, and thus insufficient to support lifecycle transparency, we propose a novel decentralized framework to serve as a common medium for interaction among open-science tools, and produces irrefutable and immutable proofs of progress that can be verified automatically.

## Introduction

In many scientific disciplines, reproducibility is the cornerstone of progress. Research that is non-reproducible due to misconducts (e.g., falsification or plagiarism) or bias not only wastes considerable amounts time and money, but also jeopardizes the credibility of the entire research community. It has been reported that only 39% of psychology articles in *Nature* could be replicated [1], and the direct cost of irreproducible preclinical research is about US$28 billion per year [2].

To conquer the reproducibility crisis [3], research communities increasingly advocate research transparency [4], which can be divided into two main levels: fuzzy and clear. The concept of *fuzzy transparency*, which is closely aligned with conventional open-access practices, consists solely of making research artifacts visible to the community so that they can be scrutinized. Here, we define an *artifact* as a constellation of elements that are needed for, yielded by, or derived during the course of a research project: data, scripts, electronic case report forms (eCRFs), internal review board (IRB) documents, benchmarks, and so on. Common recent fuzzy-transparency approaches have included sharing research artifacts, such as data or source code, alongside manuscripts.

*Clear transparency*, on the other hand, is a higher degree of transparency that appends a broader range of information to research artifacts, notably including protocols, rationales, and decisions. However, the components of clear transparency mostly focus on sharing artifacts such as data, documents, and protocols produced within a distinct research phase. As such, a holistic view of the transparency impact of research phases’ interrelationships remains elusive.

**Fig 1:**
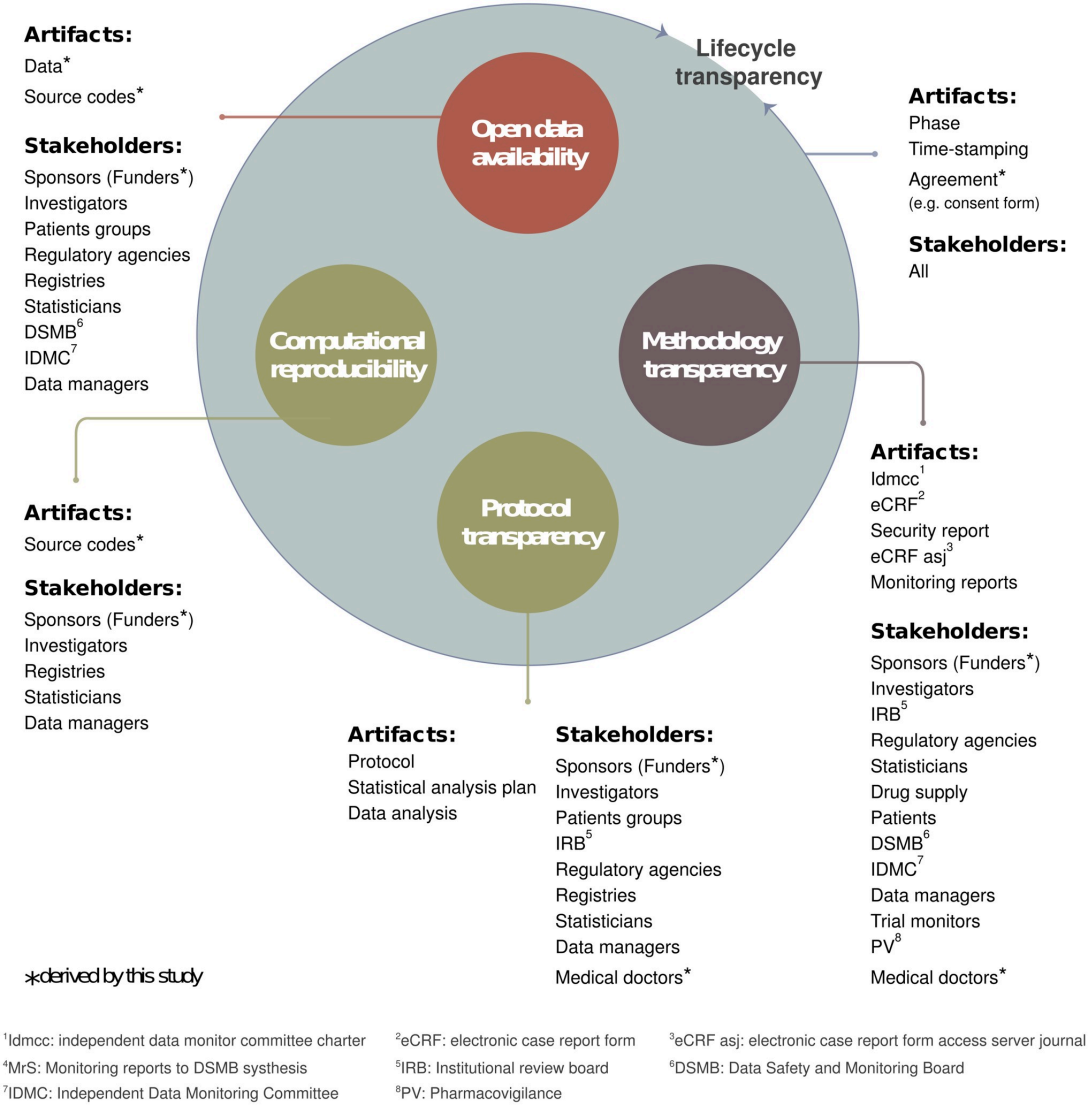
Stakeholders and associated research artifacts in clear transparency. (Note: Use case adopted from Benchoufi [5]).

In this paper, with an aim at enhancing clear transparency to conquer the reproducibility crisis, we propose *lifecycle transparency*, which captures the syntactic context of artifacts (e.g., data, manuscripts, source code) and stakeholders (researchers, participants, publishers, funders) across all phases of a research program. The syntactic context can be defined by the time, agreement, and/or dependency requirements for completing each such phase. To demonstrate the differences between different clear-transparency components, let us take as an example the clinical-trial scenario mentioned in Benchoufi’s Fig 1 [5]. As shown in our own Fig 1, lifecycle transparency can help answer questions such as “Did the PI start documenting the eCRFs after receiving patient consent?”, and thus has the potential to greatly reduce misconduct.

The achievement of lifecycle transparency will require a framework with three properties: decentralized trust, principal-investigator (PI) controllability, and automated syntax validation. Each is described in detail in its own paragraph below.

### Decentralized trust

A straightforward approach to improving lifecycle transparency would be to develop a new tool capable of recording all activities within and between research stages. However, creating a one-size-fits-all tool that captures every possible syntax produced in a research lifecycle—and that is trusted by every stakeholder including authors, funders, reviewers, publishers, readers, etc.—is a daunting, perhaps impossible task. Existing efforts to provide something akin to our concept of lifecycle transparency struggle to support the diverse research workflows required by distinct scientific disciplines. Moreover, even within a given discipline, researchers can rarely agree upon a single venue for submitting manuscripts and archiving their pre-prints and datasets. And, because researchers always risk losing or leaking information when documenting their research using third-party tools, it seems inappropriate to limit their freedom to choose which tools to use during particular research stages.

### PI-controllable transparency

Pushing for extreme transparency may also raise concerns, including but not limited to the fear of losing intellectual property, and that born-transparent artifacts will also lead to the fear of being scooped [6]. Hence, we argue that PIs should have control over what workflow should be transparent, when, and to whom, but that there should nevertheless be irrefutable and immutable proofs that can be verified once the PIs decide to disclose. Although journals and funders do not have the type of direct control exercised by PIs, the former can incentivize the latter by factoring the degree of transparency into their review processes.

### Automated syntax validation

Journals’ and funders’ review of transparency levels can be partially automated via automated syntax validation, which can be defined as the confirmation of topological order or of the existence of specific items. For example, before proceeding to a clinical trial of a new drug, the researcher must collect consent from each participating patient; and during the trial, s/he has to record patient data in a pre-defined eCRF [5] as such validation can be labor-intensive, automating it can greatly reduce human-resource demand as well as human error. However, because syntax correctness might depend on the status of multiple tools, automated syntax validation is not well supported by existing tools, which lack convenient and reliable means of informing one another of their statuses.

Our research objectives are to design, develop, and evaluate a mechanism for cultivating research lifecycle transparency. To achieve these three desired properties, we have designed and developed a decentralized framework called Blockchain for Lifecycle Transparency (BLT). BLT piggybacks programmable workflow onto existing open-science tools using Ethereum, a decentralized storage platform and program. The resulting framework allows research activities to be publicly verified, traced, and attributed, without requiring researchers and verifiers to agree on a common trusted authority. The framework also allows a researcher to commit to a customized workflow and obtain a proof-of-progress certification only when its conditions are fulfilled. Such proof of progress, in turn, can be used as an input at a later stage of the research lifecycle to enforce adherence to the committed-to procedure, such that the researcher can provide publicly verifiable proofs of their integrity, rather than relying on ethical codes and manual audits. To assess the feasibility of the BLT framework, we estimate the required cost of using a popular public blockchain as the BLT backend, and show that BLT can detect or mitigate various types of reproducibility threats and misconduct.

## Background and related work

### Types of research transparency

Four key components of clear transparency have been identified in the literature and in practice, although various research disciplines may emphasize them differently. The first, *open data availability*, also sometimes termed fuzzy transparency, describes the release of raw data only, without precise details of the researchers’ interpretive process or analysis. Researchers have observed substantial improvement in data availability since 2014, and this is due to the adoption of data mandates for scientific research by several countries. However, there is still room for improvement. For instance, one group of researchers found that, within a sample comprising one-third of the genome-wide association studies published between 2010 and 2017, only 13% reported the location of their complete sets of summary data [7].

The second of the four key components of clear transparency, *protocol transparency*, means ensuring that sufficient information is available for other research teams to reproduce an experiment or survey, including procedures, doses, sampling methods, and so on. However, it does not demand the disclosure of decision-making rationales. Researchers have been sharing their protocols via web services such as Bio-protocol [8], which allows users to discover others’ experiment protocols, as well as document and manage their own.

Third, *computational reproducibility* is quite similar to protocol transparency, but focuses more on computational aspects. It has arisen as a distinct category due to recommendations from members of the computer-science and computationally intensive research communities regarding the appropriate information to include when publishing computational findings, including access to codes, data, and execution details [9, 10].

Lastly, *methodology transparency* consists of providing access to information about decision-making, rationales, and plans over the course of research. Such information usually comprises scholarly evidence, arguments, and alternative approaches that were considered but not pursued. Proposed ways of improving methodology transparency have included pre-registration [11, 12], which documents the research inquiry and methodological parameters, and helps prevent publication bias and data-dredging [13]. Another example is the Open Science Framework [14], an online tool that supports both pre-registration and version control, and can therefore be used to document modifications, rationales, hypotheses, estimated samples, and preliminary versions of designs and analytic strategies.

To realize the first three of the above four key components of clear transparency, the research community has developed Transparency and Openness Promotion (TOP) best-practices guidelines [15] for journals, funders, scholarly associations, and other stakeholders in the research ecosystem. An evaluation metric for the quality of journals’ transparency policies, TOP Factor, has also recently been proposed [16].

### Blockchain-based solutions for open science

Blockchain technology provides the scientific community with opportunities to improve transparency and mitigate the reproducibility crisis. This is made possible by its decentralized and immutable features [17], as a blockchain’s permanently visible indexing, developed to keep records of financial transactions, can just as easily be utilized to keep records of scientific activity.

As of March 2020, there are a number of blockchain-based products aimed at improving the academic publishing process and easing concerns about irreproducible studies. For instance, ARTiFACTS [18] helps a research team to manage their research artifacts without a central authority. Knowbella [19] disseminates artifacts and builds direct funding channels between investors and researchers, again without a central authority. Orvium [20] contains a platform that allows its members to publish their manuscripts to a decentralized autonomous journal, i.e., an academic periodical without a designated publisher. Using Orvium, researchers can share their ideas via the blockchain, and attract investment in those ideas directly from the public, thus bypassing the budgetary constraints—both financial and procedural—of their own universities. Lastly, Pluto10 includes a blockchain-based reputation system with a double-blind peer-review system, aimed at reducing bias in current review processes. It maintains the reputation of both authors and reviewers, while ensuring their anonymity [21].

The Open Science Chain (OSC) project [22] provides a platform based on a consortium blockchain that enables researchers to prove the existence and integrity of their research data. The types of information researchers can record on OSC include basic information about the data (e.g., title, description, digest, keywords); its location, e.g., digital object identifier or uniform resource locators (URL); its funding sources; and contributors’ identities. OSC then allows any third party to verify the correctness of experiments via its web portal, thus facilitating future researchers’ development of their own work built on the stored results. Moreover, OSC, highlights changes across different states of research data, allowing its users to visualize the evolution of the research project as a whole. However, OSC lacks a mechanism to clarify the relationships among these different states.

## BLT: Blockchain for lifecycle transparency

BLT is a decentralized framework that allows existing and future open-science tools to interact with a decentralized backend—consisting of a storage platform and a computing platform—through a set of application programming interfaces (APIs). A key advantage of such a decentralized framework is that research workflows can be verified without requiring that researchers and verifiers agree upon a common trusted authority.

In our implementation, we use a *blockchain* as the decentralized backend, and *smart contracts* to define the APIs. For ease of demonstration, we also implemented a BLT client, a “bare-bones” open-science tool that directly invokes our supported APIs. In our implementation, each open-science tool corresponds to a smart contract on the blockchain. To interact with smart contracts, every user must have an Ethereum account (i.e., a public-private key pair), by which they can be uniquely identified on the blockchain. The BLT is open source and is stored with documentation in GitHub https://github.com/blockchain-open-science/BLT-project.

In this section, we first provide an overview of the BLT framework and its ecosystem, including the external applications with which it interacts.

### Overview: The BLT ecosystem

**Fig 2:**
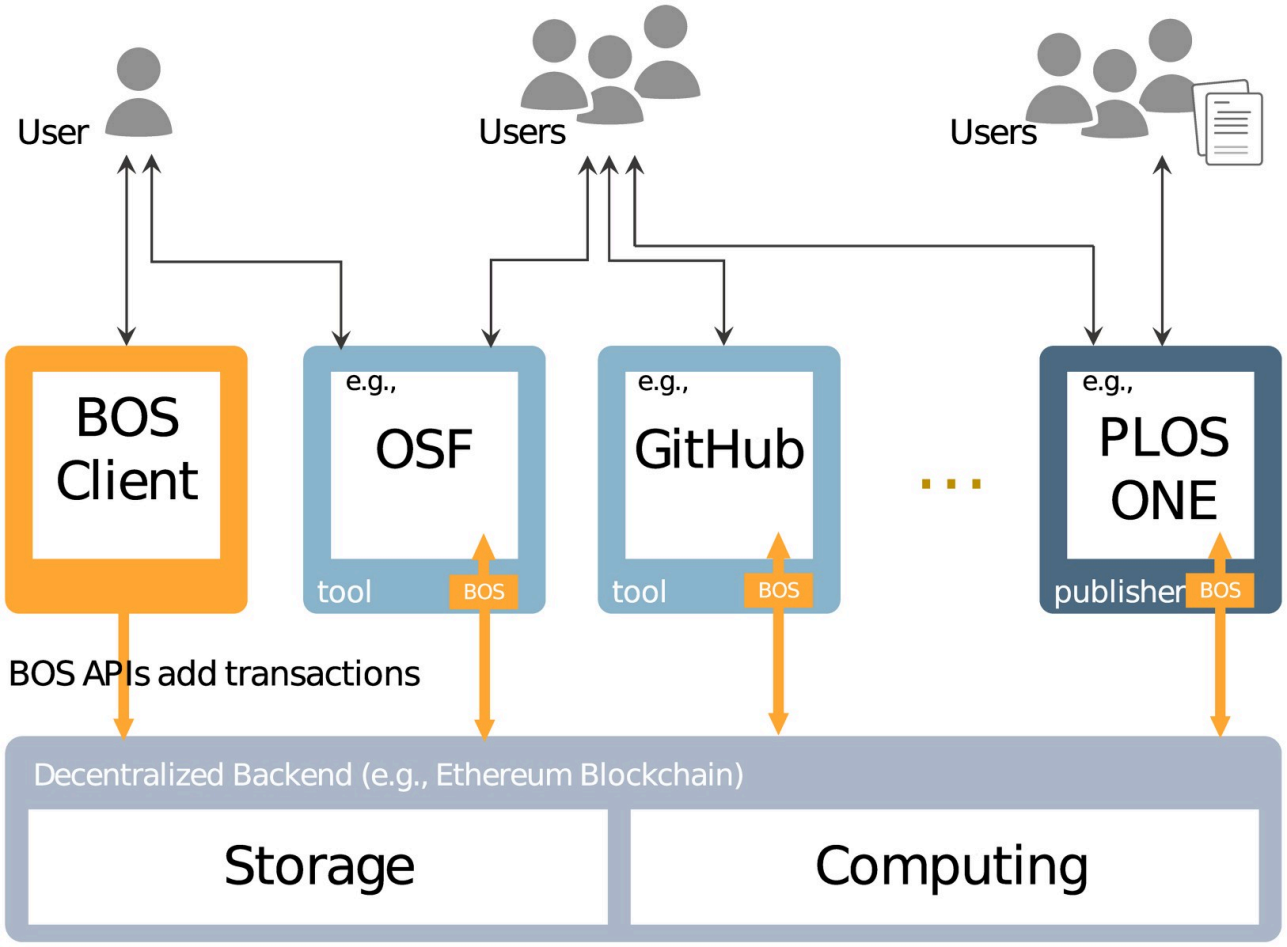
BLT ecosystem.

The BLT ecosystem, as shown in Fig 2, consists of three entity types: Decentralized Backend, Open-science Tools, and User Stakeholders. The orange-colored area indicates new components added by BLT.

#### The decentralized backend

In the BLT framework, all workflow operations (e.g., adding members to a project; creating a dependency between two research phases) are executed by the decentralized computing platform, and all transactions corresponding to the operations are stored on the decentralized storage platform. Therefore, no single root of trust is needed to ensure the integrity of a research lifecycle.

Because they are redundant and content-addressable, decentralized storage platforms like blockchain, InterPlanetary File System (IPFS), and BitTorrent are more resilient to tampering than centralized ones. Storing research records or their hash values on such decentralized platforms can also enhance the transparency of research artifacts. By contrast, when storing research records on a centralized storage platform such as Google Drive, some researchers may trust it to preserve research records without alteration, while others—including some verifiers—will not trust Google, and therefore not accept that the records are unaltered.

Decentralized computing platforms such as Ethereum ensure the correctness of computing results through redundant execution. Importantly, the code to be executed (e.g., smart contracts in Ethereum) is also stored on the blockchain to ensure immutability.

In our implementation, instead of storing the original data, only the hash of research products (e.g., data files and pure text) and the URL are stored to the blockchain. Specifically, an artifact’s hash and URL in a record are stored as state variables in the smart contract corresponding to the research project. The state variables are stored on the append-only blockchain, and thus are persistent and immutable. The state variables are declared publicly so that everyone, including the verifier, can retrieve the hash and URL of the artifacts from the blockchain. There are three key advantages to keeping only the hash and URLs: efficiency, cost reduction, and privacy. Firstly, due to the design characteristics of the Ethereum blockchain, as of December 2019 [23], the throughput of transaction data was limited to about 2.5 MB per block. Thus, it is inefficient to log a large amount of data, such as genomic data in the case of biomedical research, directly on the blockchain.

Secondly, logging a large quantity of data on a blockchain could cost a considerable sum of money. Logging the hash and URL of an artifact on the blockchain is comparatively cost-effective, and the hash provides a proof-of-existence of the artifact, based on the properties of cryptographic hash functions.

Thirdly, based on the confidentiality and privacy requirements of a project, a research group can decide when and to what extent their research artifacts should be available to the public. For example, artifacts can be publicly accessible from URLs during the whole project period; or accessible after a certain time; or only accessible to parties with a known need for such access, such as publishers. The hash value is cryptographically protected and will not leak information about the original data. However, researchers should take care to ensure that their artifacts are not corrupted or lost after logging their hashes and URLs on the blockchain, and before verification and publication.

#### The client: Open-science tools and conventional scholarly communication platforms

Existing open-science tools can access and operate on our decentralized backend through pre-defined BLT APIs. The orange arrows in Fig 2 illustrate the API interactions that create the required bridges. Existing open-science tools (such as Open Science Framework, figShare, GitHub, and the submission portal of *Nature*, *Science*, and *PLOSONE*) can easily be enabled to join the BLT ecosystem through the embedding of a piece of JavaScript code that invokes the BLT APIs. After the contract is deployed, the research group should keep the same address for it throughout the project period, and inform third-party tools of the correct address onto which their research records should be logged. During the project, researchers’ updates to the internal state of the contract are sent in the form of transactions that trigger function calls. To demonstrate the feasibility of this process, our implementation of the BLT framework includes a BLT client as a browser plugin, through which users can invoke the APIs to configure research workflows and log research activities on the decentralized backend.

On the other hand, for researchers who are not currently using any open-science tools and do not want to use our BLT client directly, they may still interact with BLT through conventional scholarly platforms that are required at different stages of research. Examples include but are not limited to the grant application system, IRB application system, enrolling records for the PhD symposiums, and Electronic Thesis and Dissertation (ETD) archives. BLT can connect digital footprints produced by not only open-science tools but these conventional scholarly information systems, therefore providing evidence for a scholar’s research journey.

### User stakeholders

As shown in Fig 2, BLT-supported open-science tools can record their users’ research-related activities on distributed storage. The BLT framework is designed to facilitate a wide array of stakeholders involved in the research cycle, including but not limited to:

- PIs (or lead authors): PIs can create projects and add members (co-authors) to them via the BLT smart contract, such that all administrative changes to the project (e.g., project creation; add or remove a member) are timestamped and logged on the blockchain. For each project, the PIs can also commit to a workflow, which they can either customize or adopt from public templates provided by journal editors, publishers, or funders.
- Contributors (or co-authors): Once added to a project by its PI, other contributors can associate their research activities (e.g., code updates; daily lab notes) or persistent digital identifiers (e.g., ORCIDs) to the project and log them on the blockchain. Via smart contracts, journal editors and funders can request the logs for further examining the project’s research activities under the special circumstance.
- Research participants: Participants, such as informants in social research or patients in clinical trials, can log their digitally signed consent forms on the blockchain, and such forms can be designated as a required input to a particular research stage within the project’s workflow.
- Journal editors: Workflow templates, created by journal editors via smart contracts, can enforce requirements such as pre-registration of study parameters, data availability (e.g., that data be uploaded to specified repositories), IRB documentation, and so forth.
- Publishers: Publishers’ requirements, such as that copyright forms be completed by the lead authors, can also be enforced via workflow template created with smart contracts.
- Funders: Funders can create workflow templates via smart contracts to enforce requirements or supplementary document such as the IRB documentation and Data Management Plans (DMP).

In the remainder of this section, we describe in more depth how users define and validate a BLT workflow that encodes the dependencies between open-science tools’ activities.

### BLT workflow

**Fig 3:**
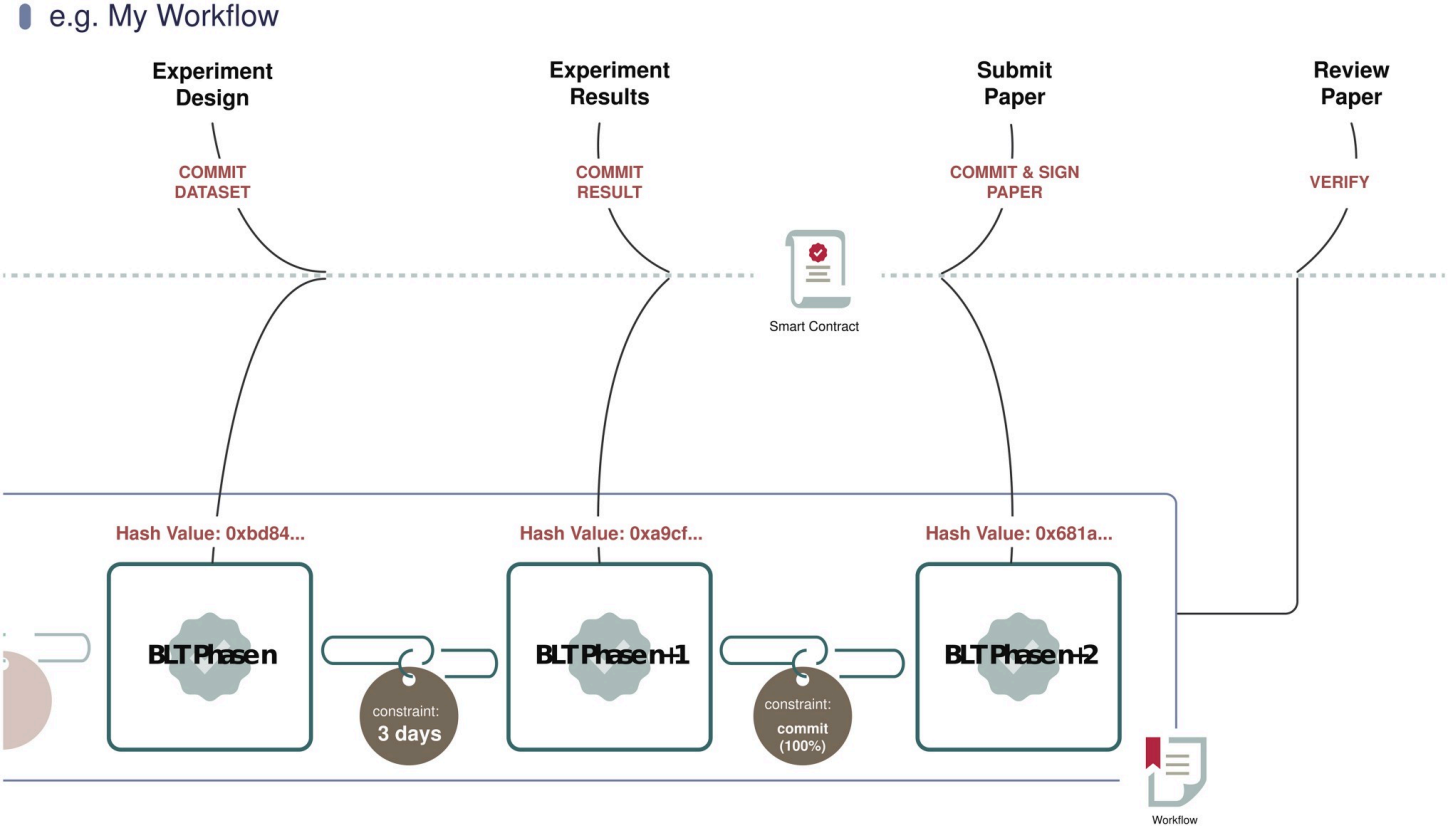
Example BLT workflow.

For ease of description, let us consider a simplified research lifecycle (Fig 3) that starts with experiment design and ends with a paper being submitted and published.

A given workflow within BLT defines a set of requirements about the syntactic structure of the records. Syntactic requirements can be specified by conditional statements (or constraints) over records’ metadata, and thus can be automatically verified via decentralized execution, e.g., Ethereum smart contracts.

One advantage of using decentralized execution here is that the correctness of the execution results does not rely on any centralized trusted authority. One example of a decentralized execution environment is Ethereum Virtual Machine, which is based on blockchain technology and uses simple re-execution and proof of work to ensure computational correctness.

A key application of verifiable workflows is the specification of domain-based workflow constraints by journals’ editorial boards or conference program committees. In that process, a contribution (e.g., an article submission) is valid only if it complies with all the specified constraints, and such compliance can be checked automatically, reducing labor requirements. A verifiable workflow in BLT should support three common types of constraints. They are:

- **Phase Constraint**: A phase constraint defines the relative order of two phases of research. For example, a researcher should perform experiments before calculating their success rates, as the reverse order would constitute misconduct. It is important to note here that a phase can either be independent, or a pre-requisite of and/or dependent upon one or more other phases. A phase that is dependent upon another is termed a child phase, and that the phase that it is dependent upon, its parent phase.
- **Time Constraint**: The existence of a time constraint implies that a specific operation needs to be finished prior to or start before either an absolute or a relative timestamp. For example, a submit operation needs to be completed before a submission deadline. Time constraints can also be augmented by phase dependency: i.e., when a child phase should occur can be constrained by when its parent phase is performed, over and above any absolute time constraints. For example, a researcher may be required to notify an affected organization at least 30 days before publishing a zero-day security vulnerability. To comply with this constraint, the researcher should upload a proof of notification first, wait the required amount of time, and then publicly disclose the vulnerability.
- **Agreement Constraint**: Lastly, an agreement constraint defines conditions based on promises made by various parties to the research. In medical data-sharing, for example, if a researcher wants to use medical data from a particular hospital (with appropriate de-identification), she should upload proof of their non-disclosure agreement before continuing to the next research phase. Another typical use case is that co-author agreements from each of the co-authors must be acquired before a paper is submitted. In our prototype, such agreements consist of digitally signed statements.

### User perspective on a BLT framework

**Fig 4:**
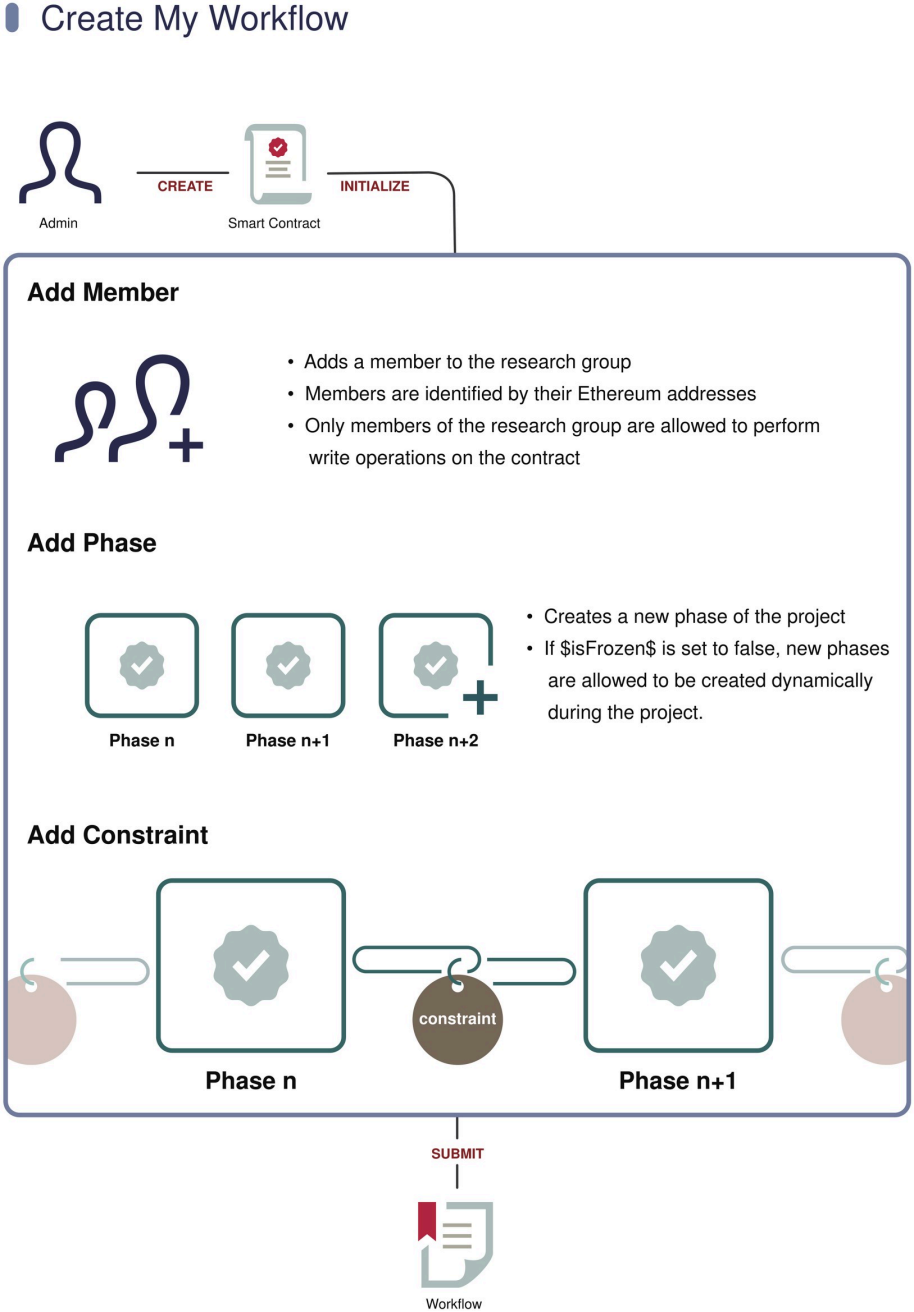
User tasks during creation of a BLT workflow.

Fig 4 presents an example of BLT-framework use in which the user is a lead PI and owner of a research project. In the BLT framework, each such project consists of a project owner, a list of members, phases, and constraints. Together, these elements comprise a workflow as introduced previously. The project owner can add members and assign different roles to each of them. The PI first divides the project into several phases, and commits the project information to the blockchain, using either the BLT Client or any supported third-party tool that invokes the BLT smart contract. A project’s information should contain the PI’s public keys and the constraints of each phase. After receiving the necessary information, the backend creates a project object and returns the project ID to the PI. Then, whenever the PI completes a phase or wishes to provide an update regarding a project milestone, s/he will send the project ID, the phase ID, and the proof of data to the blockchain. Within the decentralized backend, before changing a phase’s state, BLT will check whether all the constraints of that phase have been completed. This is enforced by the workflow, and can be publicly verified, e.g., by publishers and funders. If the phase is indeed complete, BLT will place the proof in the relevant phase, and add a tag to indicate its completion. Otherwise, it will ignore the phase-change request.

#### Smart contract template for users

In the initialization phase of a project, the research group deploys a smart contract on Ethereum blockchain. The deployed smart contracts can be templates provided by publishers or customized ones created by the research group. The contract-deployment process can be accomplished either through a publisher’s website integrated with the BLT APIs, or manually through the BLT Client. Our proposed smart-contract template consists of the functionalities listed in Table 1.

**Table 1:** Proposed smart-contract template.

Actions	Descriptions
constructor	Creates a new project, initialized with customized templates. The variable isFrozen is used to determine whether the project workflow (e.g., phase relations) can be modified during the project.
addMember	Adds a member, identified by her Ethereum address, to the research group. Only members of the research group are allowed to perform write operations on the contract.
delMember	Removes a member from the research group.
addPhase	Creates a new phase of the project. If isFrozen is set to “false”, new phases are allowed to be created dynamically during the project.
addPhaseRecord	Logs a new record to a certain phase.
commitPhase	Commits a certain phase to indicate that it is complete, and logs the committer and committed time. Once a phase is committed, new records can no longer be logged to it.
addPhaseDependency	Specifies a parent-child relationship between two phases. The child phase should not be committed until all of its parent phases are committed. This function is an implementation of the phase-dependency requirement.
addTimeConstraint	Sets a phase’s time constraint. The phase should be committed before—or, conversely, not before—a certain time. This function is an implementation of the time-constraint requirement.
calculatePhaseHash	Calculates the hash of all records in a certain phase as the phase hash. The parties who agree on the phase should sign the phase hash with their respective private keys, and send their signatures to the contract.
addAgreement	Stores a party’s signature on a phase hash, which serves as his/her/its agreement regarding that phase. This function is an implementation of the agreement requirement.
VerifyFunctions	Checks if all of a project’s phase-dependency, time, or agreement constraints have been satisfied, as of the time that this function is called.

A real-world use case can be illustrated as follows. Alice (alias) is an associate professor who runs a biomedicine research laboratory. Table 2 describes how her research activities can be actively or passively documented using BLT in more details. Note that we assume that all the tools and platforms mentioned in this example have supported and connected to BLT.

**Table 2:** Sample use case of BLT.

Research Stage	Description	Active Logging	Passive Logging
Early	1. Alice filed an IRB application for her new grant.	BLT client	IRB application system
	2. Alice met an instrument vendor and got a quote.	BLT client	–
Middle	3. Alice’s research assistant logged new data from quantitative analysis software.	Lab notebook	Internet-connected instrument
	4. Alice’s postdoc updated the open protocol in Protocols.io	–	Protocols.io
	5. Alice’s camera periodically takes a photo of the laboratory mice to record their reactions to a new drug every four hours. The hash of the photo is logged on BLT.	BLT client	–
Last	6. Alice received a rejection letter from a journal.	BLT client	Journal’s submission center

## Evaluation

In this section, to demonstrate both the practicability and conceptualization of the proposed framework, we estimate the cost of using a popular public blockchain, Ethereum, as our decentralized backend; as well as compare our functionalities against those of existing platforms; and discuss how BLT handles real-world scenarios in academia.

### Practicability evaluation: Cost

The unit that represents the computational effort that it takes to execute certain operations in Ethereum is referred to as gas. The gas required for each method in our proposed smart contract is listed in Table 3. In the Ethereum Virtual Machine execution model, the total gas used in a transaction is the sum of transaction gas and execution gas. The required amount of transaction gas depends on the quantity of transaction data and the number of non-zero bytes. Therefore, for each method calls in our smart contract, the amount required gas falls within a range, as listed in the Min and Max columns. For those methods that push elements into a global array, an initialization cost is required for the first function call, as specified in the Init column. And the calculatePhaseHash function requires additional gas for each record logged in a specific phase.

**Table 3:** Estimated gas required by BLT smart-contract methods.

Method	# of para.	Min	Max	Init
constructor	2	2,844,710	2,848,806	0
addMember	2	94,206	98,302	0
delMember	1	29,525	31,573	0
addPhase	1	92,477	94,525	0
addPhaseDependency	4	114,855	123,047	15,000
addPhaseRecord	7	154,435	168,771	15,000
addTimeConstraint	4	73,089	79,233	15,000
addAgreement	4	95,583	103,775	15,000
calculatePhaseHash*	1	45,617	47,665	0
commitPhase	1	72,378	74,426	0

* calculatePhaseHash has an additional cost of 2,728 gas per record.

Consider a project with 10 members that creates a new phase and logs 10 records every day. Assume that each phase has a dependency on the phase created the previous day, and that the whole project lasts for a year. According to our calculations, the total gas used in this project would not be more than 783,490,736 gas. In Ethereum-coin terms, if the price of gas is 1 Gwei per transaction, the entire project will cost 0.7835 Ether. At an exchange rate of US$150-350 per Ether, the maximum project cost is around US$118-274 annually. Given the current NSF’s median annualized award size in each year is about 140 thousands [24], it is at an affordable cost to document a project using BLT.

### Conceptualization evaluation: Toward better science

To test whether BLT can help improve reproducibility, we systematized threats to reproducible science (as shown in Table 4, T1 through T7) and research-misconduct types (Table 4, M1 through M6) [25]. The threat series (Tn) accounts for both sloppy science and lack of data-sharing, while the misconduct series (Mn) consists only of intentional violations of research ethics. Specifically, the conditions in T1 to T7 occur when researchers simply fail to meet the requirements of a study, such as having sufficient statistical power or controlling bias [26], whereas M1 to M6 will have much more severe consequences, once discovered.

**Table 4:** Threats to reproducible science and research-misconduct types.

Early stages of research lifecycle, e.g., idea, research design		
T1	Cognitive bias [27, 28]	In a research context, a tendency for researchers to make systematic errors in thinking and reasoning, such as unintentionally introducing bias, or having bias toward the treatment group in a clinical trial. Also includes attention bias.
T2	Low statistical power [29]	A small sample size and/or small effect can increase the likelihood of a Type II error (false negative). This, in turn, negatively influences a study’s statistical power.
M1	Plagiarism (ideas, data, results)	The taking of ideas, data, or results from others without attributing the source and claiming them as one’s own.
Middle stage of research lifecycle, e.g., data collection, quality control, analysis		
T3	Poor execution / quality-control	Poor execution of data collection, and/or poor data-quality control.
T4	Data dredging [25]	Unlike the hypothetico-deductive approach, which requires researchers to collect evidence before making a null hypothesis or other hypotheses, data dredging is the “hacking” of the data-analysis process with the aim of finding post-hoc patterns that can pass tests of statistical significance. Data dredging increases the risk of Type I errors (false positive).
M2	Fabrication	The creation or invention of nonexistent data or information related to the research.
M3	Falsification	An alteration/misrepresentation of the observed result of a scientific experiment.
Late stage of research lifecycle, e.g., reporting, publishing, data sharing		
T5	HARKing [30]	In research that purportedly utilizes a hypothetico-deductive approach, HARKing refers to hypothesizing after the results are known: i.e., introducing a post-hoc hypothesis into one’s report as if it were an a priori one.
T6	Publication bias [31]	Selective reporting and publication of only positive results. As such, it can be a combination of threats T1-T5.
T7	Lack of data-sharing [32]	Non-sharing of data can be considered a threat to the evaluation of a study’s reproducibility. Nevertheless, since data-sharing currently remains optional in many disciplines, it is not considered misconduct for our purposes.
M4	Plagiarism (writing)	The copying of others’ reported results or other written work without proper attribution.
M5	Redundant or duplicate publication	The submission of similar or identical articles to more than one venue simultaneously. A variant form, salami-slicing, means intentionally spreading the results of a single research project across multiple articles, to increase one’s overall number of publications.
M6	Ghost and Guest authorship [33]	Ghost and guest authorship refer to improper author attribution of work contribution. Specifically, ghost authorship means that a significant contributor is not listed as an author, whereas guest authorship means that a listed author does not make sufficient contribution to the work.

Table 5 presents the five features resulting from BLT use, and the types of reproducibility threats and misconduct that each such property can detect or mitigate. Each property is then discussed in its own paragraph below.

**Table 5:** Threats and misconduct types mitigated by BLT features.

Features of BLT	Threat(s) and Misconduct Type(s)
F1. Proof of existence	M1, M4, M5, M6
F2. Verifiable workflow	T5, M2
F3. Immutability of logged data	T4, T6, M3
F4. Agreement on phases	T1, T2
F5. Accessibility constraint to researchers	T7

#### F1-Proof of existence

The logged hash value of an artifact with a timestamp generated from a blockchain constitutes proof of the existence of the original data at the time of logging. This property can be used by honest researchers to prove the originality of their artifacts. For example, if a dishonest party plagiarizes a researcher’s idea and claims ownership of it, the original researcher could share the relevant documents with the public, including the hash values on the blockchain, to prove that the researcher had generated this idea at an earlier time, thereby mitigating the plagiarism of idea (M1). Proof of existence can also provide useful facts in clearing up disputes or uncertainties around ghost and guest authorship (M6) if certain authors seem to be dull or almost never participate in the course of research. For journal editors and publishers, this BLT feature is able to provide evidence for the original text and mitigate misconducts such as a dishonest party’s potential verbatim plagiarism, self-plagiarism (M4) or a duplicate publication (M5).

#### F2-Verifiable workflow

Assumed that a phase recorded on the blockchain is always synchronized with the real-world situation, the verifiable-workflow property allows definitive verification of whether a research process follows a pre-defined workflow, that is, whether all the constraints of each phase are satisfied. This property can therefore detect misconduct behaviors: for example, HARKing (T5), if the timing of the hypothesizing phase is later than that of the data-reporting phase. Data fabrication (M2) can also be flagged if the original data collection phase is later than the result writing phase.

#### F3-Immutability of logged data

Data manipulation, tampering, and falsification (M3) can all be prevented by a system in which data cannot be modified from the moment of their collection or generation to when they are logged on the blockchain. This can be achieved if, during the data-collection phase of the research, blockchain-integrated sensors are used to automatically upload all collected raw data to the blockchain and BLT backend system. The blockchain’s immutability further mitigates reproducibility threats such as data dredging (T4) and publication bias (T6), for all the “hacking” attempts or selective reporting are logged on BLT, and the logs cannot be modified or revoked.

#### F4-Agreement constraint on phases

The agreement constraint can ensure that a phase is conducted appropriately, by requiring manual examination before phase commitment. data to the blockchain and BLT backend system. For example, in the study-design phase, potential cognitive bias (T1) caused by low statistical power (T2) can be mitigated through examination by multiple verifying parties, such as peer-reviewers or research staff in the same research groups. Certain phases can also contain internal witnessing or peer review requirements in order to enhance integrity: the designated witnesses or reviewers provide approvals such that a single BLT user is not allowed to manipulate data as not being genuine (M2) or to fabricate (M3).

#### F5-Accessibility to researchers

The public blockchain allows anyone to retrieve and access the original artifacts from URIs logged in the records. This BLT feature helps promote artifact sharing possibilities, especially for research data sharing (to conquer T7). If the BLT backend is implemented with a distributed database such as IPFS, the original artifacts will also be accessible to the public permanently.

In sum, we conducted a conceptualization evaluation of BLT’s features and their connections with common threats to reproducibility as well as academic misconducts. However, because BLT is designed to automatically validate the syntactic context of the research lifecycle, BLT is not effective in mitigating semantics issues such as a poor quality of data collection (T3). More discussion will be provided in Section Discussion.

## Discussion

Lifecycle transparency improves reproducibility by revealing the high-level syntactic relationships between research artifacts and stakeholders over the course of the research lifecycle. We have presented a blockchain-based decentralized framework as a common medium of interaction among existing open-science tools, with the wider aim of fostering lifecycle transparency; and have described the various types of misconduct and other threats to reproducibility that our framework can mitigate. As a proof of concept, we implemented the framework using the Ethereum blockchain. Importantly, unlike other proposed blockchain-based solutions to the same and similar problems [18–22], our approach uses blockchain as a middleware layer to connect existing tools, rather than developing yet another tool.

We concede that the proposed BLT framework will not be a panacea for ensuring reproducibility, even if it were adopted by every open-science tool in existence. A major limitation of this work is that it focuses on the syntactic context of the research lifecycle, rather than the semantic content and its quality (i.e., T3 in Table 4). While syntax/format can be automatically validated, semantics/content still require manual validation. For example, a journal can use our framework to flag the availability status of a submitter’s data, or whether pre-registration was completed at least three months before the submission; however, it cannot be used to rate the content or dataset quality, or to assess how elaborate the pre-registration is. Likewise, our framework can help funders to flag grant applications that lack IRB approvals, but cannot help them review the content of such approvals or make suggestions about them. And it can help a journal bound by TOP guidelines to automatically verify whether a submission fulfills TOP’s Level I requirements (regarding self-declaration), Level II requirements (regarding data availability or whether pre-registration of study exists), and part of Level III requirements (regarding preserving artifacts in approved and trusted repositories), but cannot help peer reviewers or editors perform the quality checks on how a manuscript addresses the degree of design transparency specified by TOP’s Level III.

Several questions remain unanswered and require further exploration. Moving forward, we plan to collect concrete use cases from a wide range of disciplines, and synthesize generic templates from them. We plan to promote our framework and integrate it with university-level and national-level tools and repositories to achieve a large-scale, real-world evaluation. For example, a primary candidate for integration is the institutional repository and the scholar impact system (scholars.lib.ntu.edu.tw/) at our home university. This system contains the research profiles of all faculty members and students, and BLT can timestamp these research events such as adding a new publication, setting an embargo period of a thesis, receiving a research grant, etc. We would also like to conduct interviews with researchers, librarians, editors, publishers, and tool developers to refine our design and implementation.
